# Gold mining in the Amazon: the origin of the Yanomami health crisis

**DOI:** 10.1590/0102-311XEN111823

**Published:** 2023-12-22

**Authors:** Paulo Cesar Basta

**Affiliations:** 1 Escola Nacional de Saúde Pública Sergio Arouca, Fundação Oswaldo Cruz, Rio de Janeiro, Brasil.

After centuries of exploitation and violation of rights, it seems that a consensus has been reached: preserving the Amazon is the only chance for human beings to survive on the planet and a unique opportunity for Brazil to reduce historic inequities and earn international respect and credibility.

However, there is a long way to go. According to the Amazon Cooperation Treaty Organization (ACTO), there are 4,114 illegal mining sites throughout the forest. Together, they dump more than 150 tons of mercury per year in the region.

A survey conducted by the Igarapé Institute ^1^, based on 369 anti-crime operations led by the Brazilian Federal Police and environmental and judicial bodies, reveals the illicit activities affecting the Legal Amazon. The document shows that from 2016 to 2021, five environmental crimes expanded: illegal deforestation; land grabbing; illegal logging; farming with environmental liabilities; and illegal mining. In Roraima, 89% of crimes correspond to illegal mining.

Illegal mining shows multiple impacts on traditional communities. By invading ancestral territories, illegal mining results in the cutting down of vegetation, changes in the course of rivers, and the digging of large holes, changing the ecosystem and leading to widespread deforestation. As a result, native species of fauna and flora are threatened, large mammals evade the region, areas destined for shifting cultivation and collection of seasonal produce become limited, and a process of food scarcity ensues ^2^. Moreover, rivers are contaminated by mercury, poisoning fish, *tracajás*, alligators, and other animals that live in the area ^3^. As traditional foods become scarce, as there is no hunting, fishing, or other food available, food insecurity sets in.

Concomitantly, thousands of illegal miners invade indigenous lands (TI, acronym in Portuguese), impacting social organization of the communities, including abuse, aggression, and sexual violence. Moreover, once food insecurity sets in, miners distribute food in the communities, which serve as an enticement strategy and are often industrialized/ultra-processed foods, with high levels of sugar, fat, sodium, and minimal concentrations of protein ^4^. The result of this process is a double burden of nutritional deviations in the communities, in which malnourished children and older adults begin to live with young adults who suffer from overweight, obesity, diabetes, hypertension, and other diseases.

To operate the mining industry, a support network that includes heavy machinery (backhoe shovels, jet nozzles, dredges, rafts) and fuel is needed, further aggravating the social disorganization. Viscerally linked to illegal mining, alcohol, drugs, and prostitution also penetrate the area, becoming the driving force behind sexual abuse against women and children, which predisposes the spread of sexually transmitted infections. Finally, there is evidence of organized crime maintaining the illegal mining, the so-called *narcogarimpo*, and promoting drug and firearms trafficking.

This violent process alters the disease and death profile in the affected communities. Serious cases of malnutrition and the dramatic increase in cases of malaria deserve attention. Moreover, infectious respiratory illnesses such as influenza, pneumonia, tuberculosis, and COVID-19 also spread. During the pandemic, illegal mining was one of the main vectors for introducing the new coronavirus to TIs in the Amazon ^5^.

According to Matavelli et al. ^6^, the TI Kayapó and TI Mundukuru, in Pará State, as well as the TI Yanomami, in Roraima State, are the most affected by illegal mining. The devastated areas reach 11,542, 4,685, and 1,556 hectares, respectively. Based on data from MapBiomas for 1985-2020, the authors reveal that the presence of illegal mining in the Amazon has been consistent throughout the years, beginning in the late 1980s and remaining stable until the mid-2010s, when a significant and sustained increase occurred from 2016 onwards ^6^.

In the TI Yanomami, there was a peak in mining activities in the 1980s, during the first gold rush in the Amazon. After being launched by the Federal Government in 1990, the Free Jungle Operation removed tens of thousands of illegal miners from these territories. This was followed by a period of calm, with no systematic mining in the region. Since 2016, with the rise of the Far-Right to power, peace and tranquility in the territory were disrupted with a new surge of invaders. Unfortunately, the seriousness of the situation being experienced today seems to surpass that reported in the 1980s.

A study analyzing 162 Yanomami hospitalized at the Indigenous Healthcare Center (Casai, acronym in Portuguese) in Boa Vista in 1990 reported average levels of 3.61µg of mercury per gram of hair, ranging from 2.64µg/g in the Paapiú region to 5.03µg/g in the Surucucu region ^7^.

Sing et al. ^8^ conducted a study in the Catrimani River region (TI Yanomami) and found average concentrations of mercury in the participants’ blood ranging from 21.2 to 43.1µg/L in 1994. While in 1995, the average mercury concentrations in the participants’ blood ranged from 25.5 to 42.2µg/L.

It is worth remembering that the Haximu Massacre occurred in 1993 and was perpetrated by illegal miners in the TI Yanomami, resulting in the death by gunshot and machete blows of 16 indigenous people, most of them older adults, women, and children. This is considered the first genocide in Brazil.

Given this history, it can be said that there has been evidence of the impacts of illegal mining on the TI Yanomami for at least 30 years. The results are unequivocal in pointing out that mercury contamination is at the root of the health crisis.

Mercury is a metal that can undergo physical and chemical changes during its natural cycle and come in different forms: metallic mercury (used in mining to form metallic alloys with gold, forming amalgam), ionic mercury (present in environmental compartments, i.e., air, soil, water, and clouds), and organic mercury (the most toxic form, contaminating the food chain).

Burning the amalgam to separate the mercury from the gold produces fumes that are inhaled and absorbed into the bloodstream, causing damage to the lungs, brain, kidneys, and endocrine glands.

The leftover metallic mercury, separated from gold, is discharged into rivers. At the river bed, the mercury mixes with the sediment and becomes organic mercury (methylmercury). It enters the food chain, attaches itself to the muscle tissue of fish and other aquatic animals, and is later ingested as food. Methylmercury absorbed in the gastrointestinal tract spreads throughout the body and accumulates in different organs and systems, which can cause damage, expressed in different signs and symptoms, depending on the affected organ.

In the central nervous system, methylmercury can cause irreversible sensory, motor, and cognitive alterations, resulting in various damages to those affected. In adults, symptoms include hypoesthesia, tremors, abnormal gait, weakness, dizziness, seizures, vision and hearing deficits, headache, tinnitus, metallic taste in the mouth, sleep disorders, anxiety, depression, tachycardia, and hypertension.

In pregnant women, exposure to methylmercury is particularly serious, as the metal can cross the placental barrier and reach the developing fetus in the womb. Depending on the level of exposure, miscarriage or fetal death can occur. At birth, the child may have cerebral palsy, deformities, and/or congenital anomalies. Newborns may show delays in neurodevelopmental markers, such as taking a long time to sit up, crawl, take their first steps, and utter their first words. As the child ages, difficulties in playing with other children and learning can arise due to cognitive losses, which will have negative repercussions in adulthood.

Considering that the health crisis would increase with the expansion of illegal mining, in 2013, Davi Kopenawa wrote a letter to the Oswaldo Cruz Foundation (Fiocruz, acronym in Portuguese) asking for support to investigate mercury contamination in the Paapiú, Waikás-Ye’kwana, and Waikás-Aracaçá regions. From this request, the *Environment, Diversity, and Health* research group was founded to evaluate the impact of mercury exposure on the Indigenous lands of the Amazon.

In December 2014, 239 indigenous people were assessed in 19 communities across the Paapiú, Waikás-Ye’kwana, and Waikás-Aracaçá regions. Simplified clinical assessments were conducted, focusing on women and children, from whom anthropometric measurements were taken ^9^ and hair samples collected ^10^. Different levels of exposure were revealed, with the highest concentrations of mercury being recorded in the areas closest to the mining site, in both adults and children. In Waikás-Aracaçá, where mining operations began in 2013-2014, the setting was chaotic, as more than 90% of the people assessed showed elevated levels of mercury (≥ 6.0µg/g) in their hair samples ^10^. In Waikás-Ye’kwana, approximately 30% of those investigated showed levels above 6.0µg/g. It can be said that these results were expected, given the territorial invasion ^10^.

On the other hand, the situation was different in Paapiú, where, in 2014, illegal mining was not reported. Mining caused severe impacts at the end of the 1980s. However, after the Free Jungle Operation, the region was free of mining for approximately 20 years. Therefore, it was hypothesized that Paapiú would be a control area since no recent records of mining had been found. However, almost 7% of the local population had elevated levels of mercury ^10^.

The findings of Vega et al. ^10^, analyzed in a diachronic perspective with the results of the aforementioned studies ^6^ ^,^ ^7^ ^,^ ^8^, leave no doubt that mercury is a persistent environmental contaminant, leaving an extensive legacy of negative consequences for the health of TI Yanomami population.

More recently, in October 2022, 287 indigenous people were assessed in seven communities across the upper Mucajaí River (TI Yanomami). Mercury concentrations were detected in all hair samples, including men, women, children, adults, and older adults, there was no exception. Mercury levels ranged from 0.16 to 10.20µg/g, with an average of 3.79µg/g. Only three participants had levels below 1.0μg/g, the reference dose established as safe by the U.S. Environmental Protection Agency (EPA) ^11^.

Repeatedly, the current findings reinforce the long permanence of mercury in the TI Yanomami. The devastation has threatened food security and severely affected the health of the Yanomami people, revealing the latest face of the ongoing health crisis.

In a study evaluating 75 fish samples collected at different points in the Branco River basin in Roraima State, Vasconcellos et al. ^12^ found that mercury levels reached 3.16μg/g. Mercury concentrations varied according to the species analyzed and the location of the fish. In Boa Vista, on the lower Branco River, on the Mucajaí River, and on the Uraricoera River, 25%, 45%, 53%, and 57% of the studied fish presented levels ≥ 0.5µg/g, respectively. In other words, the limits of 0.5µg Hg/g of fish, established by Brazilian Health Regulatory Agency (Anvisa, acronym in Portuguese) ^13^ for the commercialization of fish in Brazil, were extrapolated at the investigated points.

The health risk assessment attributed to fish consumption ^12^, revealed that women of reproductive age and children under five ingest amounts of mercury that exceed the safety limits recommended by the EPA by 9 to 32 times, respectively ^11^.

In summary, illegal gold mining in the TI Yanomami affects more than just the indigenous people, given that the mercury that is dumped into the rivers contaminates the fish in the region and can reach city residents, including those living in the capital city, Boa Vista.

Further alerting society, a recently released study ^14^ found that 21.3% of 1,010 samples of fish purchased in markets and street fairs across 17 municipalities in the Brazilian Amazon (including the capitals Belém, Boa Vista, Macapá, Manaus, Porto Velho, and Rio Branco) had mercury levels above the parameters established by Anvisa ^13^. The worst levels of contamination were recorded in the states of Acre, Roraima, and Rondônia. In addition to the worrying levels of mercury in the fish that is consumed by families, the authors warn that the daily intake of mercury can exceed 7 to 31 times the reference dose established by the EPA ^11^ in women of reproductive age and children aged two to four, respectively.

In 2022, after the election of President Lula and before his mandate, the environmental and indigenous agenda were highlighted by the transition team, which was formed for the government transition period. Government advisors with updated information on the issue helped the government make urgent decisions:

(i) declaration of the Public Health Emergency of National Importance (ESPIN, acronym in Portuguese) in the TI Yanomami;

(ii) revocation of *Decree n. 10,966/2022* that created the Program for Supporting the Development of Artisanal Mining (Pro-Mape, acronym in Portuguese);

(iii) withdrawal of *Bill n. 191/2020* from the Brazilian Chamber of Deputies, which would legalize mining on TIs;

(iv) suspension of the effectiveness of paragraph 4, article 39, *Law n. 12,844/2013*, which provided for the presumption of good faith in the gold trade, by the Brazilian Federal Supreme Court;

(v) decision by the Brazilian Federal Revenue requiring electronic invoices to be issued for all transactions involving the purchase and sale of gold;

(vi) revitalization of the Action Plan for the Prevention and Control of Deforestation in the Legal Amazon (PPCDAM, acronym in Portuguese).

However, serious threats still loom over ancestral territories in the Amazon.

On May 30, 2023, the Brazilian Chamber of Deputies approved the *Bill n. 490/2007* (*Marco Temporal*), which to modifed procedures for the demarcation of TIs, requiring interested parties to prove their presence in claimed areas before October 5, 1988. The Federal Senate maintained its understanding, approving *Bill n. 2,903/2023* with a new number, on September 9, 2023. In turn, President Lula sanctioned *Law n. 14,701/2023* with 34 vetoes, on October 20, 2023, arguing that the Legislative’s initiative goes against the public interest and is unconstitutional “for usurping original rights” already provided for in the *Federal Constitution*. However, the fact that it was approved in both houses reveals that part of the legislative branch remains willing to promote agribusiness, industry, and mining to the detriment of original rights, favoring the illegal occupation of Union lands and environmental crimes in the Amazon.

On the one hand, it is essential to value the measures promoted by the current government. In the TI Yanomami, it is worth highlighting the creation of the Emergency Operations Committee (COE, acronym in Portuguese); the establishment of the Indigenous Health Reference Center in the Surucucu region; assistance to children and older adults with severe cases of malnutrition and malaria; distribution of food and medicines; and the beginning of the removal of illegal miners, with the support of the Brazilian Ministry of Justice.

On the other hand, we must remember that the government did not take structured action to combat illegal mining in the TI Kayapó and TI Munduruku. Another crucial point is the need to overcome historical inequalities and infrastructure deficits that affect the populations (indigenous and non-indigenous) living in the Amazon. The lack of essential public services is notorious, such as availability of drinking water, basic sanitation, access to medium and high complexity healthcare, formal education, employment, and income. In addition, limitations in transportation and communication equipment continue to affect everyone who lives in the region.

Indigenous representatives, nongovernmental organizations, social movements, universities, and research institutes must work to ensure that truthful information (based on the best scientific evidence) is disseminated to society. This will allow the nation to make better choices in the next elections and demand action from the authorities to stop the devastation immediately. Resuming the demarcation of TIs and guaranteeing the sovereignty of these territories is essential.

The preservation of the Amazon must become a global and national priority, occupying the central position in all contemporary debates! In addition to stopping the devastation immediately, reforestation is needed! It is essential to end illegal mining and eradicate the impacts of mercury contamination in Brazil. By implementing inclusive public policies and recognizing the importance of forests, it is possible to create sustainable development alternatives that are socially acceptable, environmentally appropriate, and economically viable for local populations.

Finally, the *Belém Declaration*, a document drawn up by the Amazon Alliance to Combat Deforestation during the Amazon Summit held in August 2023, emphasizes that ancestral knowledge must be valued and underscores the importance of indigenous peoples. These principles are crucial to the sovereignty of the Amazon and the promotion of sustainable development throughout Latin America.
